# Temperature-mediated biosynthesis of the phytotoxin phaseolotoxin by *Pseudomonas syringae* pv. phaseolicola depends on the autoregulated expression of the *phtABC* genes

**DOI:** 10.1371/journal.pone.0178441

**Published:** 2017-06-01

**Authors:** Selene Aguilera, Ariel Alvarez-Morales, Jesús Murillo, José Luis Hernández-Flores, Jaime Bravo, Susana De la Torre-Zavala

**Affiliations:** 1 Laboratorio Integral de Investigación en Alimentos. CONACYT-Instituto Tecnológico de Tepic, Tepic, Nayarit, México; 2 Departamento de Ingeniería Genética, Centro de Investigación y de Estudios Avanzados (CINVESTAV), Irapuato, Guanajuato, México; 3 Departamento de Producción Agraria, Universidad Pública de Navarra, Pamplona, Spain; 4 Laboratorio Integral de Investigación en Alimentos. Instituto Tecnológico de Tepic, Tepic, Nayarit, México; 5 Instituto de Biotecnología, Facultad de Ciencias Biológicas, Universidad Autónoma de Nuevo León, Monterrey, Nuevo León, México; Universite Paris-Sud, FRANCE

## Abstract

*Pseudomonas syringae* pv. phaseolicola produces phaseolotoxin in a temperature dependent manner, being optimally synthesized between 18°C and 20°C, while no detectable amounts are present above 28°C. The Pht cluster, involved in the biosynthesis of phaseolotoxin, contains 23 genes that are organized in five transcriptional units. The function of most of the genes from the Pht cluster is still unknown and little information about the regulatory circuitry leading to expression of these genes has been reported. The purpose of the present study was to investigate the participation of *pht* genes in the regulation of the operons coded into the Pht cluster. We conducted Northern blot, *uidA* fusions and reverse transcription-PCR assays of *pht* genes in several mutants unable to produce phaseolotoxin. This allowed us to determine that, in *P*. *syringae* pv. phaseolicola NPS3121, genes *phtABC* are essential to prevent their own expression at 28°C, a temperature at which no detectable amounts of the toxin are present. We obtained evidence that the *phtABC* genes also participate in the regulation of the *phtD*, *phtM* and *phtL* operons. According to our results, we propose that PhtABC and other Pht product activities could be involved in the synthesis of the sulfodiaminophosphinyl moiety of phaseolotoxin, which indirectly could be involved in the transcriptional regulation of the *phtA* operon.

## Introduction

**Fig 1:**
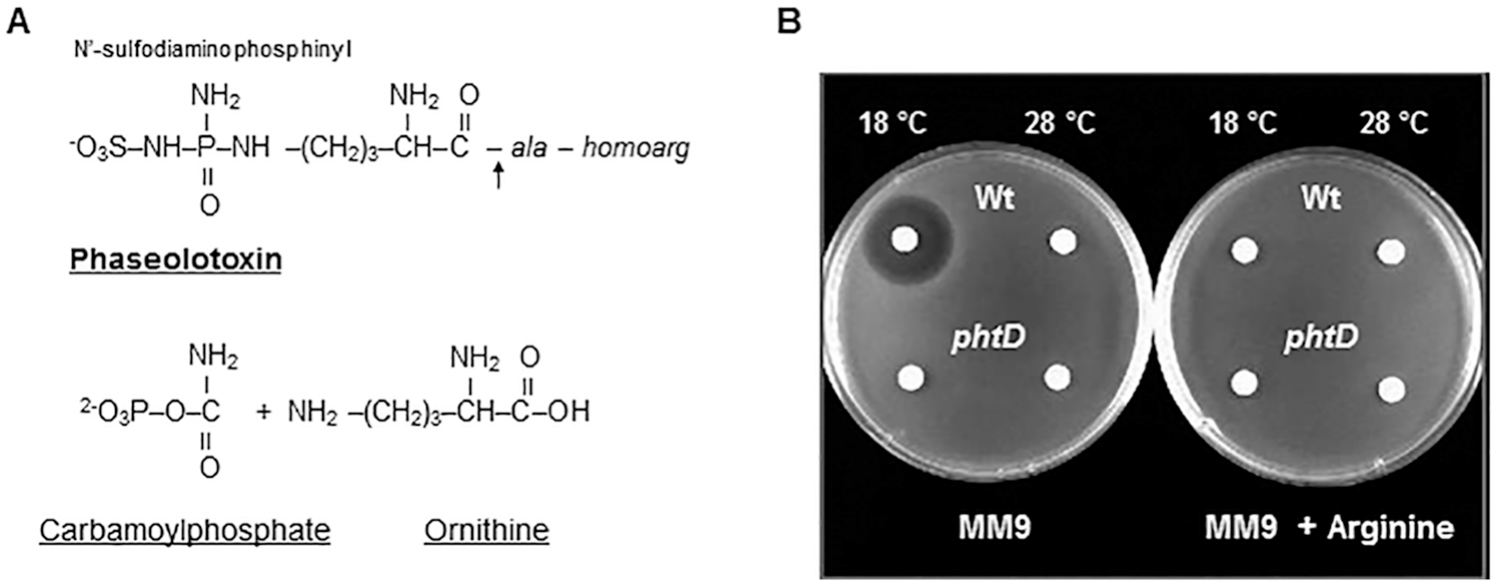
Phaseolotoxin production by *P*. *syringae* pv. phaseolicola strains. (A) Structure of phaseolotoxin (top), which is cleaved by plant peptidases (arrow) to release octicidin, with indication of the inorganic moiety (N’-sulfodiaminophosphinyl). Octicidin is a transition state analog, structurally similar to carbamoylphosphate and ornithine (bottom), substrates of OCTase during biosynthesis of citrulline. (B) Evaluation of the production of phaseolotoxin by the wild type strain NPS3121 and mutant 3121phtD using the *E*. *coli* growth inhibition assay.

**Fig 2:**
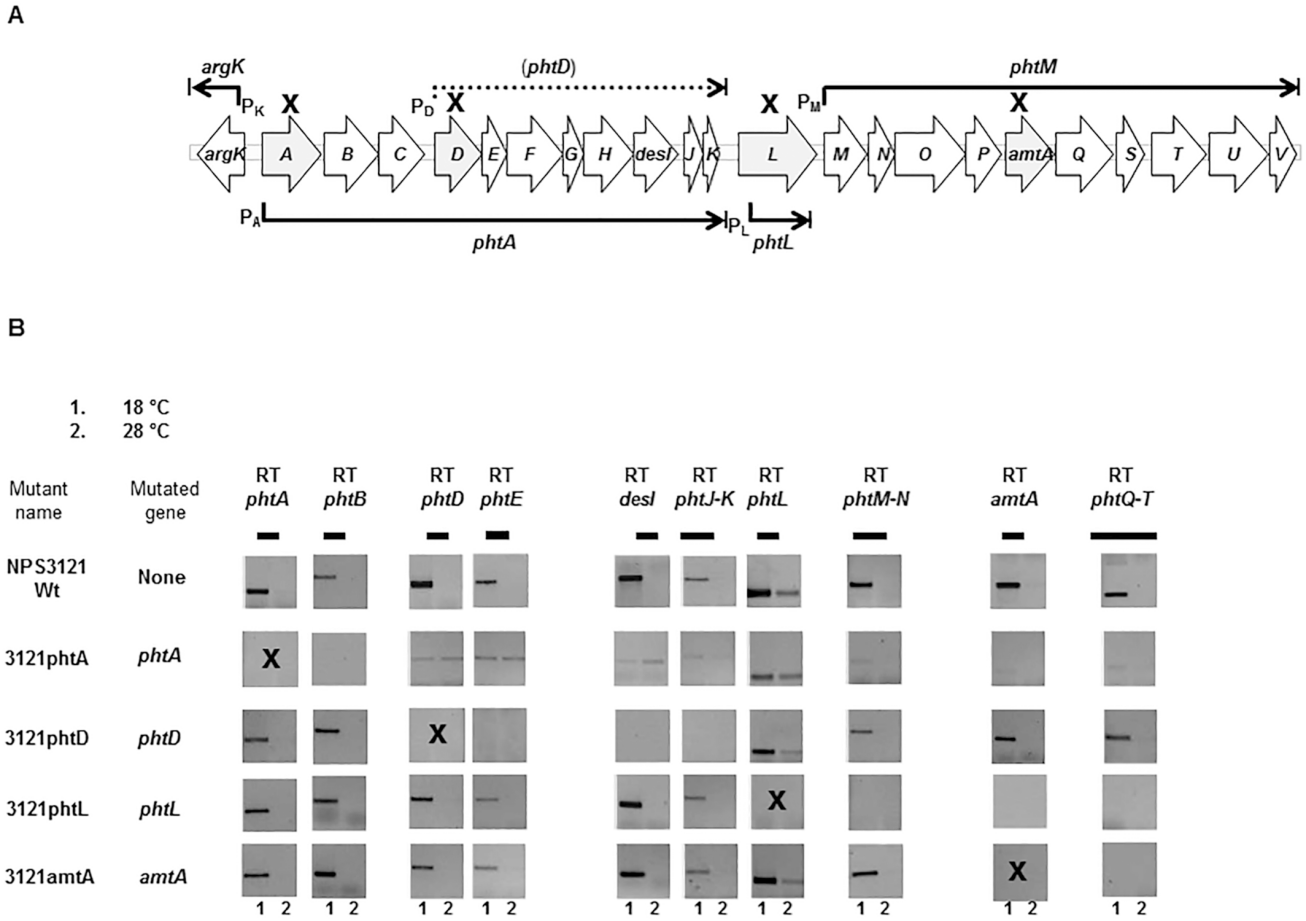
Reverse transcription-PCR of the Pht cluster of *P*. *syringae* pv. phaseolicola NPS3121 and mutants. (A) Graphic representation of the Pht cluster of *P*. *syringae* pv. phaseolicola NPS3121. Genes are represented by arrows that indicate the direction of transcription. The Pht cluster contains five transcriptional units, including two monocistronic (*argK* and *phtL*) and three polycistronic (*phtA*, *phtD* and *phtM)* [18]. Mutated genes are indicated by an X over the corresponding gene. (B) Analysis by RT-PCR of the transcriptional pattern of the Pht-cluster in the wild type strain NPS3121 and derivative mutants. Pictures show the RT-PCR product corresponding to each analyzed locus, as indicated by the black bars, separated by electrophoresis on agarose gels; mutated genes are indicated by an X over the corresponding picture of the RT-PCR gel. Numbers under the pictures represent the temperature at which expression was assayed: 1 = 18°C; 2 = 28°C.

Some strains of *Pseudomonas syringae* pv. phaseolicola and *P*. *syringae* pv. actinidiae, as well as *P*. *syringae* pv. syringae strain CFBP3388, produce phaseolotoxin, an unspecific toxin that inhibits the biosynthesis of arginine and polyamines [1–3]. Phaseolotoxin is composed of two readily identifiable moieties: one organic, an L-ornithyl-alanyl-homoarginine tripeptide, and another inorganic, *N*^*δ*^*-N´*-sulfodiaminophosphynil (Fig 1A). The toxin is cleaved by plant peptidases to generate octicidin, which is composed of the inorganic moiety joined to ornithine and is the predominant form of the toxin in infected tissues (Fig 1A). Octicidin is a transition state analog, similar to the substrates carbamoylphosphate and ornithine during biosynthesis of citrulline by the enzyme ornithine carbamoyltransferase [1, 4–6]. At relatively low temperatures (18°C to 22°C), this antimetabolite phytotoxin is produced, while no detectable amounts are present above 28°C to 30°C [7–9]. Phaseolotoxin inhibits the enzymes ornithine carbamoyltransferase (OCTase; EC 2.1.3.3) and ornithine decarboxylase (ODC; EC 4.1.1.17), which participate in the arginine biosynthetic pathway and in the biosynthesis of polyamines, respectively [10, 11]. *P*. *syringae* pv. phaseolicola is insensitive to the effect of its own toxin due to the presence of a phaseolotoxin-resistant OCTase (ROCT) [12, 13]. The *argK* gene encodes the ROCT [14, 15], which is produced under conditions leading to phaseolotoxin synthesis [16, 17]. The gene cluster involved in the biosynthesis of phaseolotoxin (Pht cluster) contains 23 genes that are organized in five transcriptional units (Fig 2A), two monocistronic and three polycistronic. In previous functional study where 15 of the 23 genes of the Pht cluster were mutagenized, only 11 of these genes lead to a Tox− phenotype whereas the four remaining mutants exhibited low levels of phaseolotoxin production [18–20]. The function of most of the genes from the Pht cluster is unknown, and only *argK*, *amtA*, *desI*, *phtL*, *phtQ* and *phtU*, which code for ROCT, an L-arginine:lysine amidinotransferase, a fatty acid desaturase, a potential pyruvate phosphate dikinase/phosphoenolpyruvate synthase, and two ATP grasp family peptide ligases, respectively, are undoubtedly involved in phaseolotoxin synthesis [14, 15, 20–24]. Additionally, a putative nonribosomal peptide synthetase (gene PSPPH_4550), located outside the Pht cluster, is also necessary for phaseolotoxin production [25].

Phaseolotoxin is produced at relatively low temperatures, allowing studies on the thermoregulation of genes involved in phaseolotoxin biosynthesis [26]. The working model for the induction of genes involved in the synthesis of phaseolotoxin in *P*. *syringae* pv. phaseolicola NPS3121 proposes that the temperature downshifts are sensed through an unknown mechanism, and then the signal is processed and transduced to an effector molecule that will act upon the repressor of phaseolotoxin biosynthesis genes [19]. At 28°C, genes involved in phaseolotoxin synthesis are negatively regulated by a repressor molecule that might bind to DNA motifs that have previously been postulated to be involved in thermoregulation [26, 27]. When temperature is downshifted to 18°C, the signal is processed and relieves repression mediated by the repressor; as a result, genes required for phaseolotoxin synthesis are actively expressed [19].

The regulatory circuitry controlling expression of the Pht cluster is apparently very complex and depends on the activity of several regulatory genes, differentially affecting its five transcriptional units. Phaseolotoxin production in *P*. *syringae* pv. phaseolicola NPS3121 requires the activity of the global regulators GacS/GacA [25] and IHF (Integration Host Factor), which binds to the promoter region of the *phtD* operon exerting a negative effect on the expression of this operon [28]. In addition, the expression of the *phtM* operon depends on the activity of gene *phtL*, also included in the Pht cluster [18]. Furthermore, genes *phtABC*, located into the Pht cluster, participate in the transcriptional repression of gene *argK* at temperatures not permissive for phaseolotoxin biosynthesis (28°C) [29]. This repression is mediated by a protein present in both toxigenic and nontoxigenic strains of *P*. *syringae* and requires the coordinated participation of the *phtABC* products in order to carry out an efficient repression of gene *argK* [29].

In this study, we investigate the participation of *pht* genes in the regulation of the operons coded into the Pht cluster. To this end, we conducted transcriptional analyses of *pht* genes in several mutants unable to produce phaseolotoxin.

## Results

### Phenotype of *phtD*^-^ mutant

To study the role of the diverse *pht* genes in their own regulation, we decided to evaluate the transcriptional activity of the Pht cluster in mutant strains in genes representing the five Pht transcriptional units. Polar mutants in genes *phtA*, *phtL* and *amtA* were already available and shown not to produce phaseolotoxin (Table 1) [18, 21]. Therefore, we disrupted gene *phtD* by insertion of a GUS-Km^r^ cassette (Table 1) thereby causing a polar mutation preventing the expression of downstream genes. This mutant, named 3121phtD, was unable to produce phaseolotoxin as shown by the growth inhibition assay (Fig 1B).

**Table 1:** Bacterial strains and plasmids.

Strain or plasmid	Relevant characteristics^a^	Reference or source
**Bacterial strains**		
*Escherichia coli*		
DH5α	*supE44* Δ*lacU169* (Φ80*lacZ*Δ*M15*) *hsdR17 recA1 endA1 gyrA96 thi-1 relA1* Nal^r^	[30]
*P*. *syringae* pv. phaseolicola		
NPS3121	Wild type, Tox^+^	[31]
CYL233	Wild type, Tox^-^	[32]
3121phtA^b^	YNorf1P; Tc^r^; *phtA*::*tet* polar mutant of NPS3121	[18]
3121phtD	Km^r^; *phtD*::*uidA*-*aph* polar mutant of NPS3121	This study
3121phtL^b^	SAorf10P; Km^r^; *phtL*::*uidA*-*aph* polar mutant of NPS3121	[18]
3121amtA^b^	AT3; Km^r^; *amtA*::*aph* polar mutant of NPS3121	[21]
**Plasmids**		
pPphtA::GUS^c^	pYUDF1; Sm^r^ Sp^r^; InReKAb; PCR fragment in pRG960sd	[18]
pPphtD::GUS^c^	pSELF1; Sm^r^ Sp^r^; InReCD; PCR fragment in pRG960sd	[18]
pUCP20	*Pseudomonas*-*E*. *coli* shuttle vector; Ap^r^; 3.89-kb; lacZ’	[33]
pRG960sd	Sm^r^ Sp^r^; 17 kb; contains a promoterless SD-*uidA* gene	[29]
pWM6	pWM6 Kanamycin cassette	[34]
pSAK	Ap^r^; *argK* in pUCP20	[29]
pSAK-A	Ap^r^; *argK-phtA* in pUCP20	[29]
pSAK-B	Ap^r^; *argK-phtB* in pUCP20	[29]
pSAK-C	Ap^r^; *argK-phtC* in pUCP20	[29]
pSAK-AB	Ap^r^; *argK-phtAB* in pUCP20	[29]
pSAK-BC	Ap^r^; *argK-phtBC* in pUCP20	[29]
pSAK-AC	Ap^r^; *argK-phtAC* in pUCP20	[29]
pSAK-ABC	Ap^r^; *argK-phtABC* in pUCP20	[29]

^a^Resistance to antibiotics is denoted as Ap^r^ (ampicillin), Km^r^ (kanamycin), Nal^r^ (nalidixic acid), Sm^r^ (streptomycin), Sp^r^ (spectinomycin), and Tc^r^ (tetracycline).

^b^YNorf1P, SAorf10P and AT3 correspond to the original name for previously constructed mutants.

^c^pYUDF1 and pSELF1 indicate the original name for previously constructed plasmids; InReKAb and InReCD correspond to intergenic regions between *argK-phtA* operons and *phtC*-*phtD* genes, respectively.

### Reverse transcription-PCR analysis in mutants

Strains 3121phtA, 3121phtD, 3121phtL and 3121amtA contain antibiotic resistance cassettes interrupting genes *phtA*, *phtD*, *phtL* and *amtA*, respectively (Table 1). These derivative strains were used to evaluate by reverse transcription-PCR analysis (RT-PCR) the effects of these mutations on the transcription of *pht* genes at 18°C and 28°C.

In mutant 3121phtA, containing a mutation in *phtA*, we could not detect expression of gene *phtB* at 18°C or 28°C, as expected for a polar mutation because *phtA* and *phtB* belong to the same operon. Genes *phtD* to *phtK* are included in the *phtA* transcriptional unit but were shown to also be transcribed as an independent operon [18], which made us expect that their expression would be only partially affected, if at all, in a strain containing a polar mutation in *phtA*. In this strain, however, genes belonging to the *phtD* operon (Fig 2A) showed a reduced expression at both 18°C and 28°C, whereas in the wild type strain NPS3121 they were strongly expressed at 18°C but not at 28°C (Fig 2B). This indicates that a polar mutation on gene *phtA* abolishes thermoregulation of the *phtD* operon. Likewise, this mutant strain also showed a reduced expression of gene *phtL* at 18°C; since this gene was shown to regulate the expression of the *phtM* operon [18], we were not surprised to also find a significantly lowered expression of operon *phtM*. Nevertheless, we cannot discard the possibility that this effect was not caused by a reduced expression of *phtL*, but resulted from a direct regulatory effect of the *phtABC* genes.

In strain 3121phtD, containing a polar mutation on the *phtD* gene, we observed the expected abolition of the transcription of the *phtD* operon (Fig 2B), In this mutant background, the pattern of transcription of genes *phtA* and *phtB* and of the operon *phtM* were similar to that of the wild type strain NPS3121, although transcription of gene *phtL* appeared to be somewhat reduced at 18°C.

In the strain containing a polar mutation in gene *phtL*, 3121phtL, we could not detect expression of the operon *phtM* at 18°C or 28°C, in agreement with previous results showing that *phtL* was necessary for *phtM* expression [18]. In contrast, the transcription pattern of the *phtA* operon was similar to that in the wild type strain NPS3121, indicating that *phtL* does not participate in the regulation of this operon.

Finally, the polar mutation disrupting gene *amtA* (mutant 3121amtA) consequently abolished the transcription of the genes belonging to the same operon and located downstream of *amtA*, but not of those located upstream (Fig 2B). Conversely, the transcriptional pattern of the *phtL* and *phtA* operons were unaffected in the mutant 3121amtA background (Fig 2B).

Together, these results suggest that genes *phtA*, *phtB* and/or *phtC*, but not other genes belonging to the *phtA* operon or downstream of *phtP* in the *phtM* operon, are participating in the regulation of the *phtD* and *phtM* operons.

### Transcriptional activity of *uidA* fusions in mutant backgrounds

**Fig 3:**
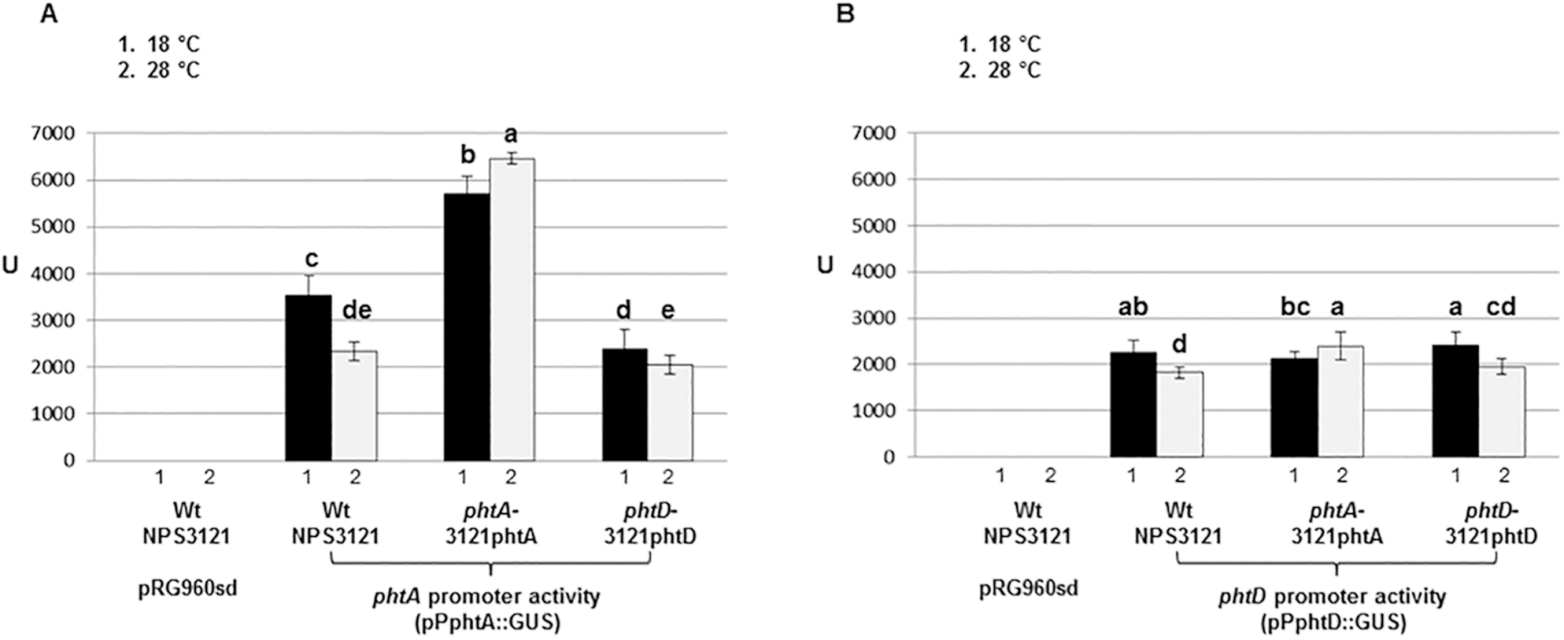
Expression of transcriptional *uidA* reporter gene fusions in *P*. *syringae* pv. phaseolicola NPS3121 and mutant backgrounds. 3121phtA and 3121phtD correspond to *phtA*^*-*^ and *phtD*^*-*^ polar mutants, whereas NPS3121 indicates the wild type strain. As negative control, NPS3121 harboring pRG960sd was used. (A) GUS activity from pPphtA::GUS, which corresponds to the promoter of *phtA* operon cloned into vector pRG960sd. (B) GUS activity from pPphtD::GUS, corresponding to the promoter of *phtD* operon cloned into pRG960sd. The small numbers under the bars represent the temperatures at which expression was assayed: 1 indicates 18°C and 2 indicates 28°C. Bars represent mean values with standard deviations; bars in each panel topped with different letters indicate means that are significantly different according to a two-way ANOVA (*P*< 0.01) followed by the Duncan’s test.

To analyze the participation of genes *phtABC* on the regulation of the *phtA* operon, we carried out a transcriptional *uidA* fusion analysis in *phtA*^*-*^ and *phtD*^*-*^ mutant backgrounds (strains 3121phtA and 3121phtD, respectively). To this end, we used plasmids pPphtA::GUS and pPphtD::GUS, which contain transcriptional *uidA* fusions to the *phtA* and *phtD* promoters, respectively (Table 1). To assess promoter activity *in trans*, these constructions were electroporated into the wild type strain NPS3121 and into the 3121phtA and 3121phtD mutants and the β-Glucuronidase (GUS) activity was quantified at 18°C and 28°C (Fig 3); nevertheless, we are aware that the levels of expression do not necessarily correlate to levels *in vivo* since the reporter gene *in trans* occurs in multiple copies [29].

The *phtA* promoter directed a higher *uidA* expression at 18°C than at 28°C in the wild type strain NPS3121, as expected, and a similar pattern was observed in the *phtD*^-^ polar mutant strain 3121phtD, although with a significantly lower expression (Fig 3A). Conversely, we observed a reversed expression pattern in the *phtA*^-^ mutant strain 3121phtA and with significantly higher levels of promoter activity than in strain NPS3121 (Fig 3A). This suggests that the activity of the *phtABC* genes participate in repressing their own transcription, with a greater repression at the nonpermissive temperature of 28°C.

The pattern of expression of the *phtD* promoter was similar to that of gene *phtA* in all strains, although with lower activity (Fig 3B), as it occurred with *phtA*, expression of *phtD* at 28°C was significantly higher in the *phtA*^-^ mutant than in the wild type (Fig 3B). Therefore, and in agreement with our RT-PCR analyses, these results suggest that the polar mutation in gene *phtA*, but not that in gene *phtD*, resulted in alleviation of the repression of the *phtA* and the *phtD* promoters at 28°C. In consequence, we focused our analysis in the participation of genes *phtA*, *phtB* and *phtC* on the regulation of the *phtA* operon.

### The products of genes *phtABC* repress their own expression in the Tox- strain *P*. *syringae* pv. phaseolicola CYL233

**Fig 4:**
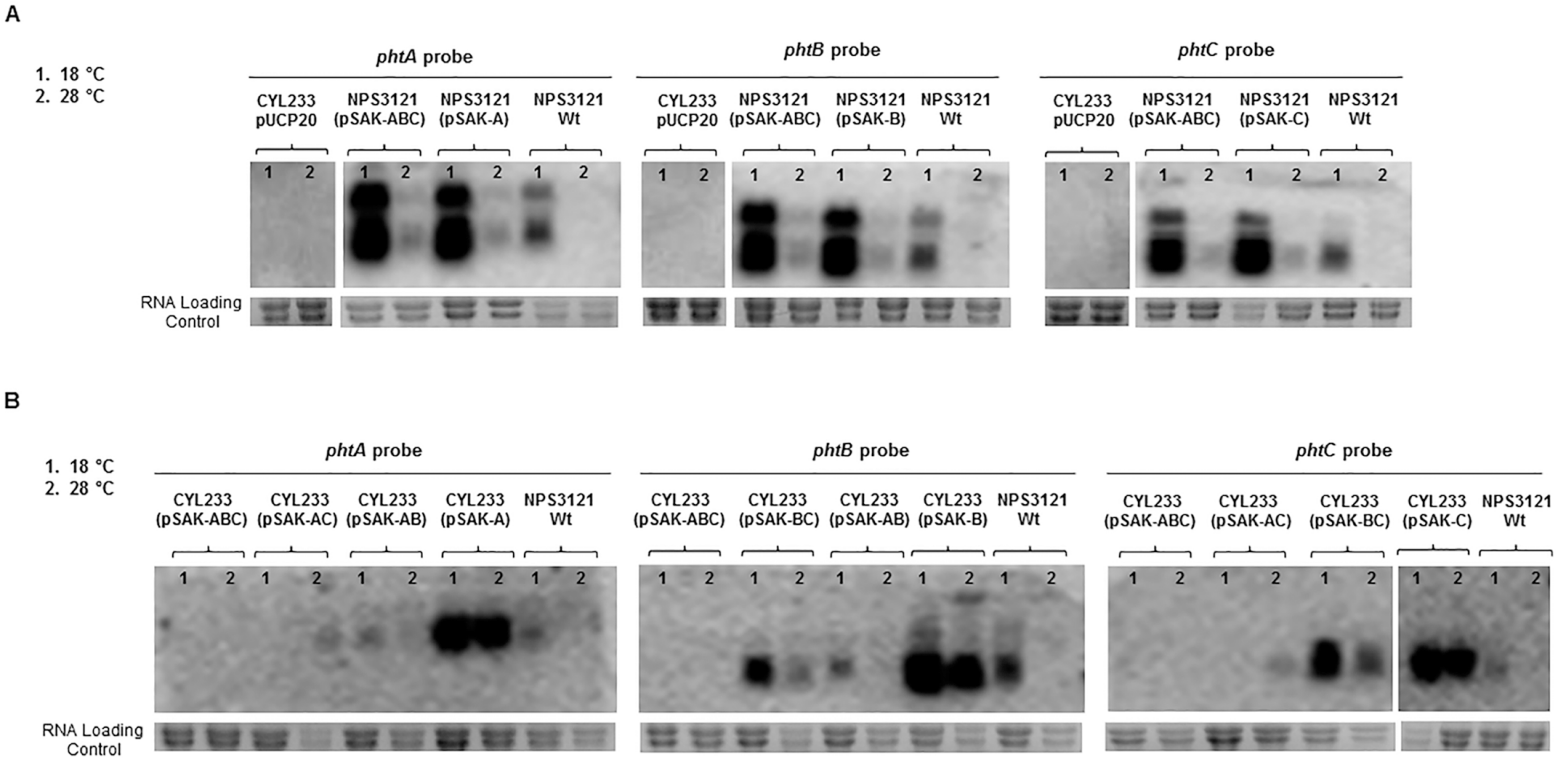
Expression of genes *phtABC* in Tox+ and Tox- strains. Northern blot hybridization of RNA isolated from *P*. *syringae* pv. phaseolicola strains NPS3121 (Tox+) and CYL233 (Tox-) containing the indicated plasmid constructs. Internal gene fragments used as hybridization probes as well as the biological source of total RNA are indicated above the gel pictures. Strain CYL233 containing the empty vector plasmid pUCP20 was used as negative control of gene expression, whereas the wild type strain NPS3121 was used as a positive control. Numbers on top of the Northern blots represent the temperatures at which expression was assayed: 1 indicates 18°C and 2 indicates 28°C.

We evaluated the participation of genes *phtABC* in the transcriptional regulation of their own promoter by Northern blot and using the *P*. *syringae* pv. phaseolicola wild type strains NPS3121 (Tox+) and CYL233 (Tox-) containing diverse constructions with these genes. Strain CYL233 is naturally unable to synthesize phaseolotoxin because it lacks the entire Pht cluster for phaseolotoxin biosynthesis [29, 32, 35, 36], and was used to discard the participation in this regulation of other genes from the Pht cluster. Plasmids pSAK-A, pSAK-B, pSAK-C, pSAK-AB, pSAK-BC, pSAK-AC, and pSAK-ABC containing genes *argK*-*phtA*, *argK*-*phtB*, *argK*-*phtC*, *argK*-*phtAB*, *argK*-*phtBC*, *argK*-*phtAC* and *argK*-*phtABC* cloned into pUCP20, respectively, were used in this study (Table 1). Clones of strains NPS3121 and CYL233 containing these constructions were grown in M9 medium at 18°C and 28°C and the *phtA*, *phtB* and *phtC* expression pattern was evaluated by Northern blot analysis (Fig 4).

In the Tox+ wild type strain NPS3121, genes *phtABC* were expressed at 18°C but not at 28°C, both when cloned individually and when cloned as an operon (Fig 4A). The low levels of *phtA*, *phtB* and *phtC* expression at 28°C in NPS3121 containing the *phtABC* constructions (Fig 4A) were not unexpected, since pSAK-A, pSAK-B and pSAK-C and pSAK-ABC are plasmids that occur in multiple copies [33] and the cloned regulation region would probably titrate the repressor. However, these genes showed a different and complex pattern of expression in the Tox- strain CYL233 (Fig 4B). When cloned individually, genes *phtA*, *phtB* and *phtC* were expressed constitutively at both tested temperatures. Clones containing the gene combinations *phtAB* or *phtBC* showed a degree of thermoregulation, resembling the situation in the wild type strain (i.e., showing strong expression at 18°C and low expression at 28°C). Unexpectedly, clones containing genes *phtAC* consistently showed a very low expression level of these two genes, and only at 28°C; we do not currently have any valid explanation for this phenomenon. Remarkably, the expression of genes *phtA*, *phtB* and *phtC* in strain CYL233 was completely abolished in clones containing the construction with the complete operon, namely pSAK-ABC (Fig 4B). This behaviour is compatible with the previously observed repression of gene *argK* by strain CYL233 containing clone pSAK-ABC [29], and indicate that genes *phtABC* participate in the repression of their own transcription and that of gene *argK*.

### Bioinformatic analysis of genes *phtABC*

In an effort to understand the functional role of *phtA*, *phtB* and *phtC* products, we conducted several analyses. While no conserved domains were found for PhtB and PhtC in an InterProScan search, PhtA belongs to the P-loop containing nucleoside triphosphate (NTP) hydrolases superfamily (InterPro SSF52540), whose members can function as kinases with very different specificities, as different kinds of motor proteins, and as batteries to drive reactions through conformational change. Further analyses in Phyre2, a protein modelling predictor [37], showed that the product of the *phtA* gene was similar to a tyrosine sulfotransferase domain (99.6% confidence), whereas PhtB and PhtC showed similarity with a cyth-like phosphatase (54.1% confidence) and a peptidyl-trna hydrolase (16.3% confidence), respectively.

Lack of conserved regulatory or DNA-binding domains and presence of domains found in proteins with enzymatic activity, indicate that the products of genes *phtABC* are not typical transcriptional regulators or DNA-binding enzymes. Rather, these genes likely exert their regulatory role by producing intermediate molecules that participate in the regulation of the Pht cluster.

## Discussion

Several efforts have been made to understand the intriguing and complex regulatory mechanism involved in phaseolotoxin synthesis and regulation. There are several gene products coded outside the Pht cluster involved in phaseolotoxin regulation: the GacA/GacS system, which is necessary for expression of operons within the Pht cluster at 18°C; the global regulator IHF, participating in the repression control of the *phtD* operon, and, finally, an unknown repressor protein able to mediate an efficient *argK* repression [25, 28, 29]. Additionally, other genes coded into the Pht cluster also participate: the gene product of *phtL* is involved in the regulation of the *phtM* operon [18, 38], whereas the products of genes *phtABC* participate in the transcriptional repression of gene *argK* at temperatures not permissive for phaseolotoxin biosynthesis [29]. The results obtained in this study indicate that in *P*. *syringae* pv. phaseolicola NPS3121, genes *phtABC* are also essential to prevent their own expression at 28°C, a temperature at which no detectable amounts of the toxin are present. Products of the *phtABC* genes lead to a very efficient repression in the nontoxigenic strain CYL233 background; however, this repression was not alleviated at 18°C, a temperature at which genes *phtA*, *phtB* and *phtC* are expressed in strain NPS3121. Conversely, *phtA*, *phtB* and *phtC* followed the wild type expression pattern, expressed at 18°C but not at 28°C, when using the same construction in the toxigenic strain NPS3121. These results may suggest that the elements necessary to de-repress the *phtA* operon at 18°C could be included into the Pht cluster; however, since these two strains are not isogenic, we cannot discard the possibility that other genes from NPS3121 are involved in this thermoregulation.

It is interesting to note that the transcriptional pattern of genes belonging to the internal operon *phtD* also appear to be regulated by the *phtABC* products, since a polar mutation in gene *phtA* resulted in a low alleviation of the repression of the *phtD* operon at the non-permissive temperature for phaseolotoxin synthesis. With respect to the regulation of this operon there is only one report demonstrating that the regulatory protein IHF binds to the promoter region of the *phtD* operon, most likely exerting a negative control on expression [28]. The regulatory connection between IHF and the PhtABC remains to be determined. Interestingly, the products of genes *phtABC* also appear to be involved in *phtL* regulation. A regulatory function for the PhtL protein has been suggested, not only regulating phaseolotoxin production [18], but also controlling the expression of genes related to iron response [38]. Functional and comparative analyses also suggested that PhtL might be involved in the biosynthesis of octicidin, participating in the formation of N-P bonds [20]. Therefore, it is feasible that octicidin might work as a toxin and also as an inducer, interacting with a regulatory protein to promote transcription of the *phtM* operon at 18°C. We observed a drastic repression of the *phtM* operon in the *phtA*^-^ mutant strain (Fig 2B), and at both 18°C and 28°C, in spite that gene *phtL* was still being transcribed. This effect, however, was not seen with the *phtD*^-^ mutant, suggesting that genes *phtABC* also participate in the regulation of the *phtM* operon. At the moment this is the first evidence about the possible regulatory connection between the *phtABC* genes and the *phtD*, *phtL* and *phtM* operons, whereby further work to investigate these regulatory mechanisms is currently under way in our laboratory.

Genes *phtABC* are transcribed divergently to *argK* and it was reported that the promoters of *phtA* and *argK* possess well-conserved -10 and -35 regions characteristic of Pribnow-type promoters [14, 18]. In a divergent transcription arrangement, usually one transcript determines a regulatory molecule and the other one determines a nonregulatory polypeptide [39]. According to our results, negative regulation of the *phtA* operon in *P*. *syringae* pv. phaseolicola NPS3121 is mediated by the products of the *phtABC* genes, as it occurs with the *argK* gene [29]. From our Phyre2 analysis, PhtB and PhtC showed similarity with a cyth-like phosphatase and a peptidyl-trna hydrolase, respectively. Additionally, PhtA was similar to a tyrosine sulfotransferase domain. Sulfotransferase activity is required to synthesize ascamycin and dealanylascamycin in *Streptomyces* sp. JCM9888. These antibiotics share an unusual 5’-O-sulfonamide moiety containing N-S bound in a similar way to the phaseolotoxin inorganic moiety [40]. Six genes (*acmABGKIW*) are hypothetically involved in 5´-O-sulfonamide formation. To explain the mechanism involved in the synthesis of 5´-O-sulfonamide, it has been proposed that sulfate ions must be activated by the formation of 3′-phosphoadenosine 5′-phosphosulfate (PAPS), which is probably catalysed by an adenylsulfate kinase (AmcB) [41]. Furthermore, sulfurylation will be catalysed by AcmB and a sulfotransferase (AcmK), which could to act upon the adenosine, utilizing PAPS as an activated sulfate donor to generate adenosine 5-sulfonate. Finally, an aminotransferase (AcmN) replace a hydroxyl group of the sulfonate with an amine group, generating the 5´-O-sulfonamide moiety [41].

According to Phyre2 analysis, the products of *phtA*, *phtS* and *phtF* genes showed similarity with a sulfotransferase, an adenylylsulfate kinase (100% confidence) and an aminotransferase (100% confidence), respectively. In agreement with this, we propose that PhtABC, PhtS and PhtF activities could be involved in the synthesis of the sulfodiaminophosphinyl moiety of phaseolotoxin, in a similar way as it occurs during the biosynthesis of ascamycin and dealanylascamycin. We are aware that PhtB and PhtC were modelled by Phyre2 with a very low level of confidence, although their involvement in the regulation of the Pht cluster in conjunction with PhtA suggests that the observed homology might be accurate and that they would also participate in the biosynthesis of the sulfodiaminophosphinyl group.

At the moment we do not have yet a satisfactory explanation about how PhtABC could be regulating their own transcription. However, it was proposed that the phaseolotoxin inorganic moiety could be acting as an indirect inducer of *pht* genes, by binding to a specific repressor molecule and removing it from its DNA target sequence [19]. Accordingly, we could hypothesize from our results that the products of genes *phtABC* participate in the synthesis of the inorganic moiety, which in turn would indirectly be involved in the transcriptional regulation of the *argK* gene [29] and the *phtA* operon.

## Materials and methods

### Bacterial strains, plasmids and media

The bacterial strains and plasmids used in this study are described in Table 1. *Escherichia coli* was cultured at 37°C using Luria-Bertani medium (LB) [30]. Strains of *P*. *syringae* pv. phaseolicola were grown at 18°C or 28°C using King´s medium B (KB) [42] or M9 medium [30]. When needed, the following supplements were added to media at the indicated final concentrations: carbenicillin, 100 μg/ml; kanamycin, 50 μg/ml; tetracycline, 10 μg/ml and spectinomycin, 50 μg/ml. All constructions were transferred to *P*. *syringae* pv. phaseolicola strains by electroporation.

### General DNA manipulations

Standard molecular biology techniques were performed as previously described [30]. Chromosomal DNA from *P*. *syringae* pv. phaseolicola was obtained as previously reported [43]. Plasmids and DNA from agarose gels were purified using Qiagen columns and kits (Valencia, CA, U.S.A.). Restriction enzymes and T4 DNA ligase were used according to the manufacturer´s instructions (Invitrogen). DNA fragments used as probes for Northern blots were labelled with [α-^32^P]dCTP by using the Rediprime random primer labeling kit (Amersham).

### Construction of a polar mutant of *phtD*

A PCR-derived amplicon containing the *phtD* gene from the phaseolotoxin gene cluster, from strain NPS3121, was cloned in the pUC19 vector; gene *phtD* was then disrupted by digestion with the restriction enzyme MscI and the insertion, in the opposite orientation to the transcription of the gene, of a 3.8 kb SmaI fragment containing the *uidA-aph* cassette from pWM6 [34]. The construct was confirmed by restriction digestion and introduced by electroporation into *P*. *syringae* pv. phaseolicola NPS3121; consequently, a polar mutant of *phtD* was obtained by the replacement of the wild type allele in the *P*. *syringae* pv. phaseolicola chromosome by a double recombination event. Kanamycin resistance was used to select for double-recombination events. The fidelity of the double recombination was confirmed by PCR analyses.

### Phaseolotoxin bioassays

Phaseolotoxin production was assayed by the *E*. *coli* growth-inhibition assay [44] as previously described [21, 45]. In every case, plates containing arginine were used as controls to confirm that growth inhibition was due to phaseolotoxin.

### GUS assays of transcriptional *uidA* fusions in mutants backgrounds

Plasmids pPphtA::GUS and pPphtD::GUS containing transcriptional *uidA* fusions to the *phtA* and *phtD* promoters, respectively, were used in this study (Table 1). These plasmids were mobilized into the *P*. *syringae* pv. phaseolicola strains NPS3121, 3121phtA and 3121phtD via electroporation. To measure β-glucuronidase (GUS) activity, strains carrying plasmids were grown in M9 medium at 18°C or 28°C to an O.D._600_ of 0.8, bacteria were then harvested by centrifugation and resuspended in GUS extraction buffer (50 mM NaHPO_4_ [pH 7.0], 10 mM disodium EDTA, 10 mM *β*-mercaptoethanol, 0.1% [wt/vol] sodium lauryl sarcosine, 0.1% [wt/vol] Triton X-100). GUS activity was determined by measuring the accumulation of 4-methylumbelliferone in a reaction mixture consisting of the bacterial lysate supplemented with 1 mM 4-methylumbelliferyl glucuronide. To terminate the reaction, 1,950 μl of 0.2 mM Na_2_CO_3_ was combined with 50 μl of the reaction mixture; then the product 4-methylumbelliferone was determined fluorometrically [46] using a VersaFluor fluorometer (Bio-Rad Laboratories, CA), to report GUS activity as specific activity (one Unit = 1 pmol of methylumbelliferone formed per minute per milligram of protein). Protein from bacterial lysates was determined by the method of Bradford [47]. As controls in these experiments, lysates from the wild type strain NPS3121, which exhibited no detectable GUS activity, were used. GUS activity is reported as the mean ± standard deviation of three repetitions, with three replicates each. Data were analyzed by two-way analysis of variance (ANOVA), with a *P*-value <0.01 considered significant, followed by Duncan’s multiple range test with a 5% significance level using the R software version 3.3.3.

### RNA extraction, reverse transcription-PCR and Northern blot analyses

RNA was isolated from cultures of *P*. *syringae* pv. phaseolicola grown in M9 medium at 18°C or 28°C to an O.D._600_ of 0.8. Total RNA was extracted from cells using TRIzol reagent as recommended by the supplier (Invitrogen) and any possible residual genomic DNA was removed from the samples by digestion with RNAse-free DNAse (Invitrogen). For reverse transcription-PCR analysis, the RNA was checked for integrity in a denaturing agarose gel and used for reverse transcription (RT) and PCR using the SuperScript One-Step Kit (Invitrogen). A list of primers used in these analyses is in Table 2. Controls used for each set of primers were: 1) PCRs without the reverse transcription step to confirm the absence of DNA; 2) RT-PCRs without RNA templates to detect any contaminating DNA/RNA; 3) PCRs using chromosomal DNA as template to ensure primer fidelity, and 4) the amplification of a portion of the 23S ribosomal RNA operon using suitable primers, as an internal control of the reaction. The RT reaction was performed at 50°C for 40 min, followed by PCR amplification at 94°C for 2 min for 1 cycle; 94°C for 35 s, 58°C for 30 s, 72°C for 2 min for 25 cycles; 72°C for 15 min for 1 cycle.

**Table 2:** Primers used in this study.

Primer Name	Primer sequence	Gene/locus
P25156	GCAAAAACGAAAACACCAGGCT	*phtA*
Ptx1I5c	ATCGCGCTGATCCGGAAAGG	
1415	CTTGTCGAGGCGAATAGCGTGTGG	*phtB*
P23243	CCAAAACGGCTATTTAACAACGGC	
P1101	AGTTCGGCGGTCCATGGTAT	*phtD*
P1102	ACCCGACATCGAAAGCAGCG	
P16881	TCAACAACATCCACGGGCAT	*desI*
G720	GATATCGCAGCAACACCCATAAAAC	
1253	TTGCGACGGATCCCTACGCCAATCTATCGAC	*phtJ-K*
L100116phtL	GATATCCGACGTACATTCGATAACCC	
FIPIA	ATCTTTATACGGCCGCCGACCC	*phtL*
P12718	TACGTTGCCCGGTGTCGACA	
P12556	TCCGGTTATCGCTTCAGGTCG	*phtM-N*
P11311	GCAGTTTCTGATCTTGGGCCC	
BRL519	TTCATTCAAACCTCGCCCGTGTG	*amtA*
BRL520	TGAAAGGAGCCGCCGAAACTATTG	
1251	CTACAGCGGATCCAATAGCACCGATAGCAAG	*phtQ-T*
1243	CCAATATCCCGGGCCACTTATCTACTTTCCT	
P25156	GCAAAAACGAAAACACCAGGCT	*phtA*
Ptx1I5c	ATCGCGCTGATCCGGAAAGG	
1415	CTTGTCGAGGCGAATAGCGTGTGG	*phtB*
P23243	CCAAAACGGCTATTTAACAACGGC	
Ptx1I2c	CCACTGGCGTTGGCTTATTTCG	*phtC*
P21868	TATCCACGTCGTCATTGAGCG	
P1108	TCTGCTGTTAGGTTTAAGTG	*phtD*
P1109	AGATCCAAGAAAACCAGGTC	

For Northern blot analysis, samples of total RNA (20 μg) were denatured by treatment with formamide and separated by electrophoresis using 1.3% denaturing agarose gels. The RNA was transferred to Hybond-N+ nylon membranes (Amersham) and cross-linked by exposure to UV radiation. Hybridization was performed using NorthernMax Prehybridization/Hybridization buffer (Ambion) using internal fragments of the genes *phtA*, *phtB*, *phtC* as probes. All probes were labeled with [α-^32^P]dCTP by using the RediPrime Random Primer labeling kit. After overnight hybridization at 60°C, membranes were washed twice with 2X SSC-0.1% sodium dodecyl sulfate (1X SSC is 0.15 M NaCl and 0.015 M sodium citrate) for 5 min at room temperature, followed by a wash with 1X SSC-0.1% sodium dodecyl sulfate for 15 min at 60°C. Membranes were exposed in a Storm 860 apparatus and the resulting image was analyzed using ImageQuant version 1.1 software.

## References

[1] Mitchell RE. Isolation and structure of a chlorosis-inducing toxin of *Pseudomonas phaseolicola*. Phytochemistry. 1976;15:1941–7.

[2] Tamura K, Imamura M, Yoneyama K, Kohno Y, Takikawa Y, Yamaguchi I, et al Role of phaseolotoxin production by *Pseudomonas syringae* pv. actinidiae in the formation of halo lesions of kiwifruit canker disease. Physiol Mol Plant Pathol. 2002;60:207–14.

[3] Tourte C, Manceau C. A strain of *Pseudomonas syringae* which does not belong to pathovar phaseolicola produces phaseolotoxin. Eur J Plant Pathol. 1995;101:483–90.

[4] Mitchell RE, Johnston JS, Ferguson AR. Phaseolotoxin and other phosphosulphanyl compounds: biological effects. Physiol Plant Pathol. 1981;19:227–35.

[5] Templeton MD, Sullivan PA, Shepherd MG. The inhibition of ornithine transcarbamoylase from *Escherichia coli* W by phaseolotoxin. Biochem J. 1984;224: 379–88. 6393952 10.1042/bj2240379 PMC1144443

[6] Templeton MD, Reinhardt LA, Collyer CA, Mitchell RE, Cleland WW. Kinetic analysis of the L-ornithine transcarbamoylase from *Pseudomonas savastanoi* pv. phaseolicola that is resistant to the transition state analogue (R)-N delta-(N'-sulfodiaminophosphinyl)-L-ornithine. Biochemistry. 2005;44(11):4408–15. doi: 10.1021/bi047432x 15766270 10.1021/bi047432x

[7] Goss RW. The relation of temperature to common and halo blight of beans. Phytopathology. 1940;30:258–64.

[8] Nuske J, Fritsche W. Phaseolotoxin production by *Pseudomonas syringae* pv. phaseolicola: the influence of temperature. J Basic Microbiol. 1989;29:441–7. 2600779 10.1002/jobm.3620290713

[9] Mitchell RE. Halo blight of beans: toxin production by several *Pseudomonas phaseolicola* isolates. Physiol Plant Pathol. 1978;13:37–49.

[10] Bachmann AS, Matile P, Slusarenko AJ. Inhibition of ornithine decarboxylase activity by phaseolotoxin: implications for symptom production in halo blight of French bean. Physiol Mol Plant Pathol. 1998;53(5–6):287–99.

[11] Ferguson AR, Johnston JS. Phaseolotoxin-chlorosis, ornithine accumulation and inhibition of ornithine carbamoyltransferase in different plants. Physiol Plant Pathol. 1980:269–75.

[12] Ferguson AR, Johnston JS, Mitchell RE. Resistance of *Pseudomonas syringae* pv phaseolicola to its own toxin, phaseolotoxin. Fems Microbiol Lett. 1980;7:123–5.

[13] Staskawicz BJ, Panopoulos NJ, Hoogenraad NJ. Phaseolotoxin insensitive ornithine carbamoyltransferase of *Pseudomonas syringae* pv. phaseolicola: basis for immunity to phaseolotoxin. J Bacteriol. 1980;142:720–3. PubMed Central PMCID: PMC294059. 7380807 10.1128/jb.142.2.720-723.1980 PMC294059

[14] Hatziloukas E, Panopoulos NJ. Origin, structure, and regulation of *argK*, encoding the phaseolotoxin resistant ornithine carbamoyltransferase in *Pseudomonas syringae* pv. phaseolicola, and functional expression of *argK* in transgenic tobacco. J Bacteriol. 1992;174:5895–909. PubMed Central PMCID: PMC207126. 1522066 10.1128/jb.174.18.5895-5909.1992 PMC207126

[15] Mosqueda G, Van den Broeck G, Saucedo O, Bailey AM, Alvarez-Morales A, Herrera-Estrella L. Isolation and characterization of the gene from *Pseudomonas syringae* pv. phaseolicola encoding the phaseolotoxin-insensitive ornithine carbamoyltransferase. Mol Gen Genet. 1990;222:461–6. 2274044 10.1007/BF00633857

[16] Jahn O, Sauerstein J, Reuter G. Detection of two ornithine carbamoyltransferases in a phaseolotoxin-producing strain *Pseudomonas syringae* pv. phaseolicola. J Basic Microb. 1985;25:543–6. 10.1002/jobm.3620250821 4087158

[17] Jahn O, Sauerstein J, Reuter G. Characterization of two ornithine carbamoyltransferases from *Pseudomonas syringae* pv. phaseolicola, the producer of phaseolotoxin. Arch Microbiol. 1987;147:174–8. 3592910 10.1007/BF00415280

[18] Aguilera S, Lopez-Lopez K, Nieto Y, Garciduenas-Pina R, Hernandez-Guzman G, Hernandez-Flores JL, et al Functional characterization of the gene cluster from *Pseudomonas syringae* pv. phaseolicola NPS3121 involved in synthesis of phaseolotoxin. J Bacteriol. 2007;189:2834–43. PubMed Central PMCID: PMC1855804. doi: 10.1128/JB.01845-06 17237165 10.1128/JB.01845-06 PMC1855804

[19] Lopez-Lopez K, Hernandez-Flores JL, Cruz-Aguilar M, Alvarez-Morales A. In *Pseudomonas syringae* pv. phaseolicola, expression of the *argK* gene, encoding the phaseolotoxin-resistant ornithine carbamoyltransferase, is regulated indirectly by temperature and directly by a precursor resembling carbamoylphosphate. J Bacteriol. 2004;186:146–53. PubMed Central PMCID: PMC303443. doi: 10.1128/JB.186.1.146-153.2004 14679234 10.1128/JB.186.1.146-153.2004 PMC303443

[20] Chen L, Li P, Deng Z, Zhao C. Ornithine transcarbamylase ArgK plays a dual role for the self-defense of phaseolotoxin producing *Pseudomonas syringae* pv. phaseolicola. Sci Rep. 2015;5:12892 PubMed Central PMCID: PMC4530439. doi: 10.1038/srep12892 26256666 10.1038/srep12892 PMC4530439

[21] Hernandez-Guzman G, Alvarez-Morales A. Isolation and characterization of the gene coding for the amidinotransferase involved in the biosynthesis of phaseolotoxin in *Pseudomonas syringae* pv. phaseolicola. Mol Plant-Microb Interact. 2001;14:545–54. 10.1094/MPMI.2001.14.4.545 11310742

[22] Hatziloukas E, Panopoulos NJ, Delis S, Prosen DE, Schaad NW. An open reading frame in the approximately 28-kb tox-*argk* gene cluster encodes a polypeptide with homology to fatty acid desaturases. Gene. 1995;166:83–7. 8529898 10.1016/0378-1119(95)00569-5

[23] Zhang YX, Patil SS. The *phtE* locus in the phaseolotoxin gene cluster has ORFs with homologies to genes encoding amino acid transferases, the AraC family of transcriptional factors, and fatty acid desaturases. Mol Plant-Microb Interact. 1997;10:947–60. 10.1094/MPMI.1997.10.8.947 9353942

[24] Arai T, Kino K. A novel L-amino acid ligase is encoded by a gene in the phaseolotoxin biosynthetic gene cluster from *Pseudomonas syringae* pv. phaseolicola 1448A. Biosci Biotechnol Biochem. 2008;72:3048–50. doi: 10.1271/bbb.80439 18997422 10.1271/bbb.80439

[25] De la Torre-Zavala S, Aguilera S, Ibarra-Laclette E, Hernandez-Flores JL, Hernandez-Morales A, Murillo J, et al Gene expression of Pht cluster genes and a putative non-ribosomal peptide synthetase required for phaseolotoxin production is regulated by GacS/GacA in *Pseudomonas syringae* pv. phaseolicola. Res Microbiol. 2011;162:488–98. doi: 10.1016/j.resmic.2011.04.010 21527339 10.1016/j.resmic.2011.04.010

[26] Rowley KB, Clements DE, Mandel M, Humphreys T, Patil SS. Multiple copies of a DNA sequence from *Pseudomonas syringae* pathovar phaseolicola abolish thermoregulation of phaseolotoxin production. Mol Microbiol. 1993;8(3):625–35. 8326870 10.1111/j.1365-2958.1993.tb01606.x

[27] Rowley KB, Xu R, Patil SS. Molecular analysis of thermoregulation of phaseolotoxin-resistant ornithine carbamoyltransferase (*argK*) from *Pseudomonas syringae* pv. phaseolicola. Mol Plant-Microb Interact. 2000;13(10):1071–80. 10.1094/MPMI.2000.13.10.1071 11043468

[28] Arvizu-Gomez JL, Hernandez-Morales A, Pastor-Palacios G, Brieba LG, Alvarez-Morales A. Integration Host Factor (IHF) binds to the promoter region of the *phtD* operon involved in phaseolotoxin synthesis in *P*. *syringae* pv. phaseolicola NPS3121. BMC Microbiol. 2011;11:90 PubMed Central PMCID: PMC3112066. doi: 10.1186/1471-2180-11-90 21542933 10.1186/1471-2180-11-90 PMC3112066

[29] Aguilera S, De la Torre-Zavala S, Hernandez-Flores JL, Murillo J, Bravo J, Alvarez-Morales A. Expression of the gene for resistance to phaseolotoxin (*argK*) depends on the activity of genes *phtABC* in *Pseudomonas syringae* pv. phaseolicola. PLoS One. 2012;7(10):e46815 PubMed Central PMCID: PMC3466206. doi: 10.1371/journal.pone.0046815 23056465 10.1371/journal.pone.0046815 PMC3466206

[30] Sambrook J, Fritsch EF, Maniatis T, editors. Molecular cloning: a laboratory manual. 2nd ed. New York: Cold Spring Harbor; 1989.

[31] Peet RC, Lindgren PB, Willis DK, Panopoulos NJ. Identification and cloning of genes involved in phaseolotoxin production by *Pseudomonas syringae* pv. "phaseolicola". J Bacteriol. 1986;166(3):1096–105. PubMed Central PMCID: PMC215237. 3011734 10.1128/jb.166.3.1096-1105.1986 PMC215237

[32] Rico A, Lopez R, Asensio C, Aizpun MT, Asensio-S-Manzanera M, Murillo J. Nontoxigenic strains of *Pseudomonas syringae* pv. phaseolicola are a main cause of halo blight of beans in Spain and escape current detection methods. Phytopathology. 2003;93(12):1553–9. doi: 10.1094/PHYTO.2003.93.12.1553 18943619 10.1094/PHYTO.2003.93.12.1553

[33] West SE, Schweizer HP, Dall C, Sample AK, Runyen-Janecky LJ. Construction of improved *Escherichia*-*Pseudomonas* shuttle vectors derived from pUC18/19 and sequence of the region required for their replication in *Pseudomonas aeruginosa*. Gene. 1994;148:81–6. 7926843 10.1016/0378-1119(94)90237-2

[34] Metcalf WW, Wanner BL. Construction of new beta-glucuronidase cassettes for making transcriptional fusions and their use with new methods for allele replacement. Gene. 1993;129(1):17–25. 8335256 10.1016/0378-1119(93)90691-u

[35] Murillo J, Bardaji L, Navarro de la Fuente L, Fuhrer ME, Aguilera S, Alvarez-Morales A. Variation in conservation of the cluster for biosynthesis of the phytotoxin phaseolotoxin in *Pseudomonas syringae* suggests at least two events of horizontal acquisition. Res Microbiol. 2011;162(3):253–61. doi: 10.1016/j.resmic.2010.10.011 21187143 10.1016/j.resmic.2010.10.011

[36] Bardaji L, Echeverría M, Rodríguez-Palenzuela P, Martínez-García PM, Murillo J. Four genes essential for recombination define GInts, a new type of mobile genomic island widespread in bacteria. Sci Rep. 2017;7:46254 doi: 10.1038/srep46254 28393892 10.1038/srep46254 PMC5385486

[37] Kelley LA, Mezulis S, Yates CM, Wass MN, Sternberg MJ. The Phyre2 web portal for protein modeling, prediction and analysis. Nat Protoc. 2015;10(6):845–58. PubMed Central PMCID: PMC5298202. doi: 10.1038/nprot.2015.053 25950237 10.1038/nprot.2015.053 PMC5298202

[38] Gonzalez-Villanueva L, Arvizu-Gomez JL, Hernandez-Morales A, Aguilera-Aguirre S, Alvarez-Morales A. The PhtL protein of *Pseudomonas syringae* pv. phaseolicola NPS3121 affects the expression of both phaseolotoxin cluster (Pht) and Non-Pht encoded genes. Microbiol Res. 2014;169:221–31. doi: 10.1016/j.micres.2013.05.002 23806843 10.1016/j.micres.2013.05.002

[39] Beck CF, Warren RA. Divergent promoters, a common form of gene organization. Microbiol Rev. 1988;52(3):318–26. PubMed Central PMCID: PMC373147. 3054465 10.1128/mr.52.3.318-326.1988 PMC373147

[40] Langley DB, Templeton MD, Fields BA, Mitchell RE, Collyer CA. Mechanism of inactivation of ornithine transcarbamoylase by Ndelta -(N'-Sulfodiaminophosphinyl)-L-ornithine, a true transition state analogue? Crystal structure and implications for catalytic mechanism. J Biol Chem. 2000;275(26):20012–9. doi: 10.1074/jbc.M000585200 10747936 10.1074/jbc.M000585200

[41] Zhao C, Qi J, Tao W, He L, Xu W, Chan J, et al Characterization of biosynthetic genes of ascamycin/dealanylascamycin featuring a 5'-O-sulfonamide moiety in *Streptomyces* sp. JCM9888. PLoS One. 2014;9(12):e114722 PubMed Central PMCID: PMC4257720. doi: 10.1371/journal.pone.0114722 25479601 10.1371/journal.pone.0114722 PMC4257720

[42] King EO, Ward MK, Raney DE. Two simple media for the demonstration of pyocyanin and fluorescin. J Lab Clin Med. 1954;44(2):301–7. 13184240

[43] Chen WP, Kuo TT. A simple and rapid method for the preparation of gram-negative bacterial genomic DNA. Nucleic Acids Res. 1993;21(9):2260 PubMed Central PMCID: PMC309503. 8502576 10.1093/nar/21.9.2260 PMC309503

[44] Staskawicz BJ, Panopoulos NJ. A rapid and sensitive assay for phaseolotoxin. Phytopathology. 1979;69:663–6.

[45] Sawada H, Suzuki F, Matsuda I, Saitou N. Phylogenetic analysis of *Pseudomonas syringae* pathovars suggests the horizontal gene transfer of *argK* and the evolutionary stability of *hrp* gene cluster. J Mol Evol. 1999;49(5):627–44. 10552044 10.1007/pl00006584

[46] Jefferson RA, Kavanagh TA, Bevan MW. GUS fusions: beta-glucuronidase as a sensitive and versatile gene fusion marker in higher plants. EMBO J. 1987;6(13):3901–7. PubMed Central PMCID: PMC553867. 3327686 10.1002/j.1460-2075.1987.tb02730.x PMC553867

[47] Bradford MM. A rapid and sensitive method for the quantitation of microgram quantities of protein utilizing the principle of protein-dye binding. Anal Biochem. 1976;72:248–54. 942051 10.1016/0003-2697(76)90527-3

